# Grooming as a secondary behavior in the shrimp *Macrobrachium rosenbergii* (Crustacea, Decapoda, Caridea)

**DOI:** 10.3897/zookeys.457.6292

**Published:** 2014-11-25

**Authors:** Lauren N. VanMaurik, Jennifer L. Wortham

**Affiliations:** 1 University of South Florida, Department of Integrative Biology; 2 University of Tampa, College of Natural and Health Sciences

**Keywords:** Grooming, aquaculture, *Macrobrachium rosenbergii*

## Abstract

The giant freshwater prawn, *Macrobrachium rosenbergii*, is a large shrimp extensively used in aquaculture whose grooming behaviors were analyzed in this study. *Macrobrachium rosenbergii* exhibits three unique male morphotypes that differ in their behavior, morphology and physiology: small-clawed males (SM), orange-clawed males (OC) and blue-clawed males (BC). The largest and most dominant males, BC males, are predicted to have significantly different grooming behaviors compared to females and the other two male morphotypes. These BC males may be too large and bulky to efficiently groom and may dedicate more time to mating and agonistic interactions than grooming behaviors. Observations were conducted to look at the prevalence of grooming behaviors in the absence and presence of conspecifics and to determine if any differences in grooming behavior exist among the sexes and male morphotypes. Significant differences in the grooming behaviors of all individuals (females and male morphotypes) were found. BC males tended to have the highest grooming time budget (percent of time spent grooming) while SM males had a relatively low grooming time budget. The grooming behaviors of the male morphotypes differed, indicating while these males play distinct, separate roles in the social hierarchy, they also have different grooming priorities. The conditions in which *Macrobrachium rosenbergii* are cultured may result in increased body fouling, which may vary, depending on the grooming efficiencies and priorities of these male morphotypes. Overall, grooming behaviors were found to be a secondary behavior which only occurred when primary behaviors such as mating, feeding or fighting were not present.

## Introduction

### Behavioral hierarchy

A behavioral hierarchy occurs among certain behaviors which are deemed essential to an organism. Ranking of behaviors by individuals is necessary when an organism is in conflict situations such as foraging (Davis et al. 1974), fighting a predator or conspecific (Karplus et al. 1987), or mating (Liske and Davis 1986). These behaviors are normally deemed primary behaviors and are usually considered high in a behavioral hierarchy as they are evolutionarily important for reproductive fitness and survival situations. Secondary behaviors should occur when primary behaviors are not critical. An example of a secondary behavior is body grooming which is a behavioral adaptation to fouling pressures. Grooming removes fouling agents and has been hypothesized, but not tested, to be a secondary behavior (Bauer 1989). These secondary behaviors may be inhibited when more vital behaviors are beneficial to survival. Therefore, grooming would decrease when primary behaviors are more pressing (Bauer 1989, 2013).

The environment that an organism inhabits is also an important factor in the behavioral decision making process. Organisms in resource-limiting environments or social situations (i.e. competition, mating, agonistic interactions) should prioritize those behaviors with the greatest resource profitability (primary behaviors, i.e. searching for food or mates) before other subordinate behaviors (Brown 1986, Shettleworth 2009). Thus, it is hypothesized that only when the organism is in a situation where environmental pressures are not critical will secondary behaviors like grooming take place (Bauer 1989). Organisms adjust their behavioral schedule to best accommodate their needs. Grooming is important in daily maintenance activities of organisms to ensure that the body is free of fouling that may impede important primary actions such as foraging for food, reproduction, and avoiding predation (VanMaurik and Wortham 2011).

### Grooming behaviors in animals

Grooming is a behavior for removing fouling debris and organisms from body surfaces (Bauer 1977, Felgenhauer and Schram 1978). It is a common behavior seen in many animals including primates (Dunbar 1996), birds (Cotgreave and Clayton 1994), fish (Bshary and Schaffer 2002), insects (Hlavac 1975) and crustaceans (Bauer 1977, 2004, 2013, Felgenhauer and Schram 1978, 1979, Martin and Felgenhauer 1986). Although grooming occurs in both terrestrial and aquatic systems, the former have relatively fewer fouling pressures than the latter (Holmquist 1985). Aquatic biota are constantly bathed in a water medium where fouling can be particularly detrimental if the surrounding water is laden with sediment or fouling organisms (Bauer 2004).

### Grooming in Crustaceans

Autogrooming in crustaceans is important for removing macro- and microscopic fouling organisms, debris, sedimentation, and algae from body surfaces (Bauer 1981). Grooming in crustaceans prevents fouling of structures utilized in reproduction (pleopods; Bauer 1979), respiration (gills; Bauer 1998, 1999), sensory reception (antennae; Bauer 1977, 1978), as well as displays and movements by jointed appendages (Bauer 1981, 1989). Fouling of these structures may result in decreased efficiency of respiration and sensory reception along with decreased ability to mate, brood offspring and fight competitors (Bauer 1977, 1978, 1979, 1998, 1999). Crustaceans have an exoskeleton, jointed appendages and soft body parts (i.e. gills) that are periodically molted which can remove some fouling from the outer body surfaces. Although molting removes most fouling agents from the body, the intermolt period may be lengthy, especially for older individuals that are normally larger and molt less frequently and therefore have a decreased ability to rid themselves of fouling (Skinner et al. 1985).

Grooming behaviors have been studied for many crustacean groups, especially in decapod crustaceans such as penaeid and caridean shrimps (Bauer 1977, 1978, 1979, 1981, 1989, 1999, 2004, 2013, Felgenhauer and Schram 1978, 1979, VanMaurik and Wortham 2011), brachyuran crabs (Bauer 1981, Pearson and Olla 1977), anomuran crabs (Martin and Felgenhauer 1986), lobsters (Schmidt and Derby 2005), crayfishes (Bauer 1998, 2002), and stomatopods (Wortham 2008). Despite the phylogenetic relatedness of these groups, there is considerable variability in the grooming behaviors and morphology of decapods. This observed behavioral variation in decapods (crabs, shrimps, lobsters) is thought to accommodate their unique morphologies. As a result, the amount of time devoted to grooming varies among crustacean groups (i.e. grooming time budget).

### Study organism

**Figure 1.**
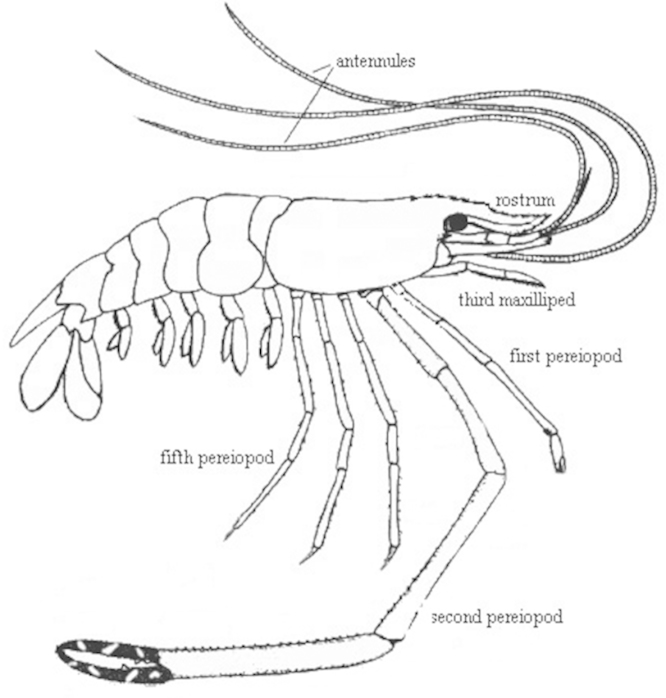
Generalized morphology of *Macrobrachium rosenbergii*. (Diagram modified from Short 2004).

The genus *Macrobrachium* has over 240 species (De Grave et al. 2009, De Grave and Fransen 2011) and has long, robust chelipeds (second pereopods, Fig. 1), especially in large adult males (Wowor et al. 2009). The giant freshwater prawn, *Macrobrachium rosenbergii* (Crustacea: Decapoda: Caridea), is a caridean shrimp native to rivers of Southeast Asia but has been introduced to most continents for aquaculture. Of the 1.2 million tons of globally cultured shrimps in 2000, only 10% was caridean shrimps. But of that 10%, almost 99.9% were the shrimp, *Macrobrachium rosenbergii* (FAO 2010), with a global aquaculture economic value of $410 million, representing a significant product (New and Valenti 2000).

**Figure 2.**
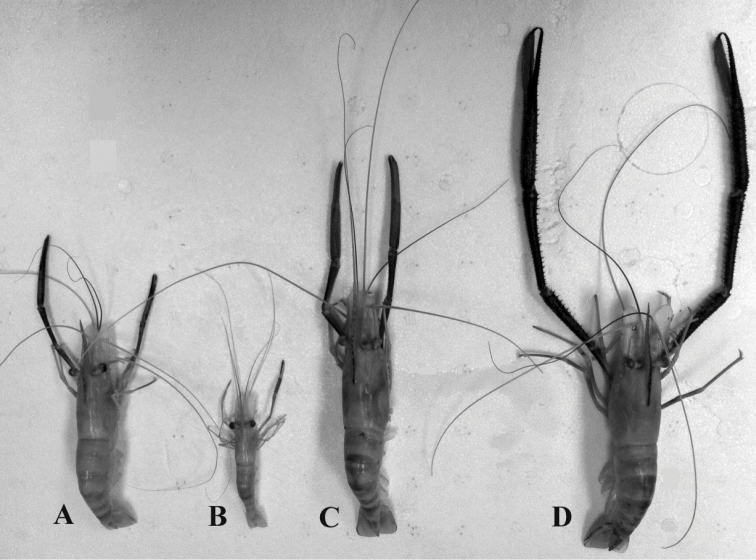
Relative size of *Macrobrachium rosenbergii* female and male morphotypes. **A** Female **B** Small-clawed (SM) male **C** Orange-clawed (OC) male **D** Blue-clawed (BC) male. Note the difference in the size of the chelipeds.

Agonistic behaviors and social structure of *Macrobrachium rosenbergii* have been extensively studied due to its use in aquaculture (Barki et al. 1991, Kuris et al. 1987, Ra’anan and Sagi 1985). This species has three distinct male morphotypes, which differ in morphology, physiology and behavior (Ra’anan and Sagi 1985, Kuris et al. 1987, Sagi and Ra’anan 1988) (Figure 2). The smallest males (SM) have small claws and are subordinate and non-territorial. The intermediate orange-clawed males (OC) are subdominant to the larger males and larger in body size and cheliped (second pereopod) length than SM males. The largest and dominant males in the population are the blue-clawed males (BC). These male morphotypes form a social hierarchy in the population and may be found within the same age class (i.e. all three morphotypes belong to the same cohort) (Kuris et al. 1987, Govind and Pearce 1993).

The three male morphotypes of *Macrobrachium rosenbergii* exhibit behavioral differences based on their position within the social hierarchy (Kuris et al. 1987). BC males may devote more time to growth of large chelipeds and body size, agonistic interactions with males, reproduction with females, protection of post-molt females, social displays, and defending territories compared to the other male morphotypes. Devotion to these behaviors may result in decreased time available for grooming. While grooming has been a reported behavior for this species, scant information is available (Ra’anan and Sagi 1985, Karplus et al. 1992).

### Objectives and hypotheses

Due to the importance of *Macrobrachium rosenbergii* in aquaculture, understanding the grooming behaviors of this species is vital for implementing ways to increase yield and growth by decreasing the potential fouling affecting the morphotypes. The most profitable size is the large BC males and development into this terminal male morphotype depends upon surviving through the SM and OC male phases. We hypothesized *Macrobrachium rosenbergii* will: (1) have similar grooming behaviors to other caridean shrimps; (2) BC males will have less time available for grooming than other males due to time dedicated to the protection of females, defense of territories and dominance behaviors; and (3) the grooming will be a secondary behavior as hypothesized by Bauer (1989). By studying the grooming behaviors of *Macrobrachium rosenbergii*, especially the male morphotypes, a key understanding of the tradeoffs between body maintenance and social dominance may be elucidated. Grooming may be an important behavior at the lower levels of the social hierarchy (i.e. SM males) but not in the dominant tier (BC males) where mating and maintaining territories are essential. Understanding how fouling and grooming behaviors vary among morphotypes may impact yield and profit, therefore, aquaculture operators have expressed interest in the grooming behaviors of the morphotypes and the possible ramifications on the survivorship of the shrimps (Craig Upstrom, Aquaculture of Texas, personal communication).

## Methods

*Macrobrachium rosenbergii* were transported overnight from Texas in April 2012 to the University of Tampa. Individual shrimps were added to labeled plastic holding containers (5.5-L), with pre-drilled holes that allowed water flow, and then placed in an 1816-liter fiberglass aquaculture tank with filtered, continuous flowing, aerated water. The individual containers reduced physical contact and agonistic interactions, ensuring that both shrimps’ appendages remained intact and death by cannibalism was eliminated. The containers allowed visual and pheromonal contact among individuals as water was able to flow through the pre-drilled holes. The three male morphotypes (SM, OC and BC males) were distinguished by morphological characteristics and correlations among mass and body measurements (Kuris et al. 1987). Shrimps were not fed on testing days; on non-testing days, they were fed shrimp pellets ad libitum. Throughout the study, shrimps were kept on a 14/10 day-night cycle, and water temperature and salinity ranged from 22–24 °C and 5–10 ppt, respectively. Shrimps were not reused in a particular set of observations, however, due to the scarcity and cost of research animals, some shrimps were reused for different sets of observations. Duplicate measurements or observations were never made.

### Statistical analyses

The grooming data were analyzed to determine if they met the criteria for parametric statistics. If normality assumptions were not met, then non-parametric statistics were used. Along with variability in individual behavior and failure to meet normality, the grooming data were analyzed using non-parametric statistics. Non-parametric statistical tests used included the Kruskal-Wallis test and the Mann-Whitney U test. Regression analyses were also used. Statistical significance was determined by p-value of less than 0.05.

### Grooming Observations

#### Solitary grooming (Observations #1)

Behavioral observations were conducted to study the grooming behaviors of *Macrobrachium rosenbergii*. The null hypotheses of no difference in relative time budgets allocated to grooming behaviors among the three male morphotypes and between sexes were tested. Individuals were tested in isolation, which helped reduce primary behaviors such as fighting and mating. Each shrimp was used once (N=94) in these solitary observations and placed into a 19-L (40 cm × 25 cm × 20 cm) aquarium with black backing and natural rocky substratum. The black backing ensured that the shrimp would not be influenced by either surrounding shrimps in other tanks or the observer. Water in the aquarium was continually filtered and frequently replaced with water from the aquaculture tanks. Shrimps were allowed to acclimate for 24-hr before testing and were not used if they had molted within seven days. Females with embryos (“eggs”) on their pleopods were not observed to control for the behavior among males and females. All grooming behaviors were recorded during the daylight cycle for 30-min using a digital recording device and then later transcribed to data sheets following the methods of VanMaurik and Wortham (2011). These data were used to determine differences in the time spent grooming and body parts groomed among the morphotypes and between the genders.

#### Social grooming (Observations #2)

The null hypothesis that all behaviors will be equally prioritized was tested. To observe how social interactions with conspecifics affect grooming behaviors, each male morphotype (SM, OC, and BC males) and females were placed in a grooming situation where shrimps could physically touch through antennular and cheliped contact (but not fight) via holes in the individual containers. The objective was to compare grooming behaviors of individuals in an environment without visual or minor physical cues (Solitary Grooming – Observations #1) to that of an environment with visual and physical cues (Social Grooming – Observations #2). These latter observations differed from the Solitary Grooming (Observations #1) because individuals in the isolated situation only had chemical contact through water with other shrimps but did not have visual or minor physical input that was present in the Social Grooming (Observations #2). Visual and minor physical contact with conspecifics was expected to reduce frequency and time allocated to grooming behaviors in these observations, since these behaviors have been predicted to be secondary. Shrimps (N=8; two shrimps of male morphotypes plus females) were observed in the aquaculture tanks in their individual containers for 15-min and their grooming behaviors were recorded. These shrimps were randomly selected from the first observations (Solitary Grooming) and observed 24-hr after being used in the first observations. The same process of recording behaviors was used as in the Solitary Grooming (Observations #1). The data collected in these social observations were extrapolated (multiplied by 2) in order to compare the data to those from the Solitary Grooming (Observations #1) (15-min × 2 = 30-min).

#### Agonistic interactions (Observations #3)

The null hypothesis that all behaviors are equally prioritized was tested to determine how agonistic interactions (primary behaviors) affect the priority of grooming behaviors of the male morphotypes (BC, OC, and SM) and females. The objective of these observations was to compare the frequency of grooming behaviors in an environment without visual cues (Solitary Grooming, Observations #1) to that of an environment with physical contact (Agonistic Interactions, Observations #3). If grooming behavior is a secondary action incurring the same energy cost as primary behavior (i.e. mating, fighting, displaying), grooming behaviors should be reduced in time and frequency during these observations compared to both the solitary and social observations (Observations #1 and #2, respectively). During these observations shrimps had physical contact with another individual in a test arena and their grooming behaviors were recorded along with all other behaviors such as swimming, antennular touching, mating, fighting and non-agonistic interactions (interactions with no aggressive behaviors). This is different from the Social Grooming (Observations #2) due to the increased level of interaction (i.e. fighting, mating possible). In Observations #3, shrimps were paired based on morphotype and sex for a total of ten different treatments; there were two trials of each treatment for a total of N=20 observations (Table 1). Following a 24-hr acclimation period within individual containers, shrimps were allowed to acclimate for 30-min in a test arena (within water table: 58 cm × 41 cm × 23 cm) without any contact. For the subsequent 30-min, the shrimp’s behaviors were recorded while in it’s individual container. Most shrimps had ample room to walk around within its individual container. After the observations were completed, the number of behaviors and types of behaviors were tabulated. Shrimps used in this Agonistic Interaction (Observations #3), were randomly selected from the Solitary Grooming (Observations #1) and were observed after a minimum of 24-hr acclimation period within individual containers.

**Table 1.** Experimental design of Agonistic Interactions (Observations #3), listing the ten treatments and the number of trials for each treatment. BC = blue-clawed males; OC = orange-clawed males; SM = small-clawed males; F = females.

Treatment	Individual #1	Individual #2	Sample Size
Treatment 1	BC	BC	2
Treatment 2	BC	OC	2
Treatment 3	BC	SM	2
Treatment 4	BC	F	2
Treatment 5	OC	OC	2
Treatment 6	OC	SM	2
Treatment 7	OC	F	2
Treatment 8	SM	SM	2
Treatment 9	SM	F	2
Treatment 10	F	F	2

## Results

### Grooming observations

#### Solitary grooming observations (Observations #1)

##### Appendages: overall

**Figure 3.**
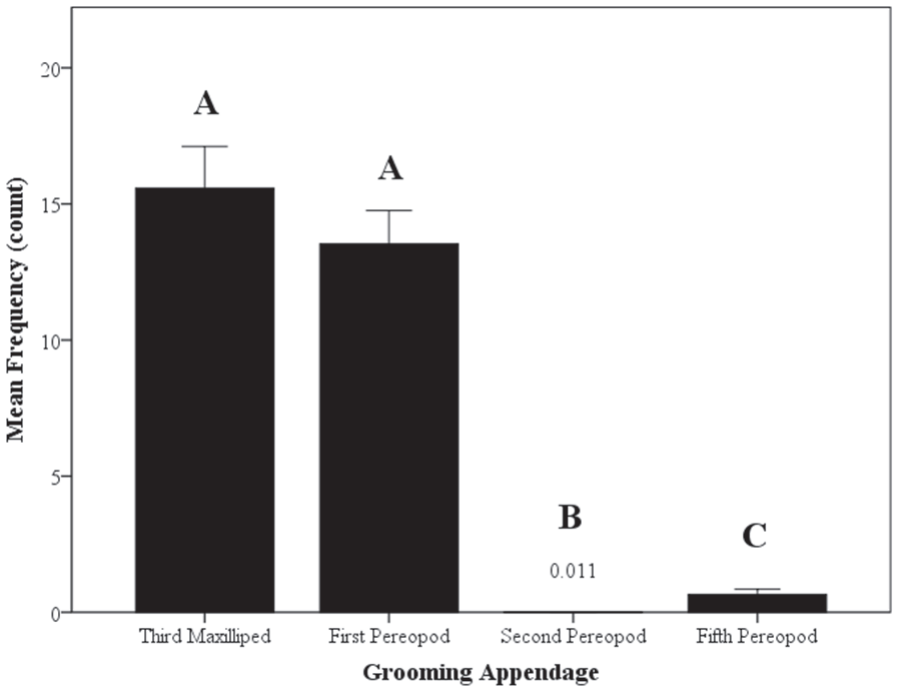
Mean frequency of use of grooming appendages of *Macrobrachium rosenbergii* (N=94) in 30-min time period (mean ± SE). Note: different letters indicate significant differences among use of appendages.

Four appendages were observed actively grooming the body: third maxilliped (M3), first pereopod (P1), second pereopod (P2), and fifth pereopod (P5) (Fig. 1). The largest, most cumbersome appendage is the chelate P2 which was rarely used in grooming (Fig. 3). The smaller, more mobile chelate P1 appendage is better suited to access harder-to-reach and tighter spaces, and it is one of the appendages that are used more frequently in grooming (Fig. 3). The P1-carpal propodal brush also sweeps over the A2 in a quick grooming action, often assisted by the M3. The M3 is frequently used to scrape anterior areas of the body such as other appendages, antennae and antennules; each individual M3 grasps the appendage or antenna/antennule of one side and scrapes from the proximal to the distal end of the groomed appendage. The P5 appendage, or the fifth walking leg, is not chelate like P1 or P2 and was used to scrape posterior parts of the body such as the telson, abdomen or pleopods (Fig. 3). The M3 and P1 were used approximately the same in grooming (Kruskal-Wallis, H=219.88, P<0.001; Mann-Whitney U, z=0.40, P=0.69; Fig. 3) and were the most frequently used grooming appendages (Mann-Whitney U, z=10.24–11.45, P<0.001; Fig. 3). The P2 and P5 appendages were used much less frequently than M3 and P1, but the P5 was used significantly more than the P2 (Mann-Whitney U, z=-4.26, P<0.001; Fig. 3).

##### Appendages: Morphotypes

**Figure 4.**
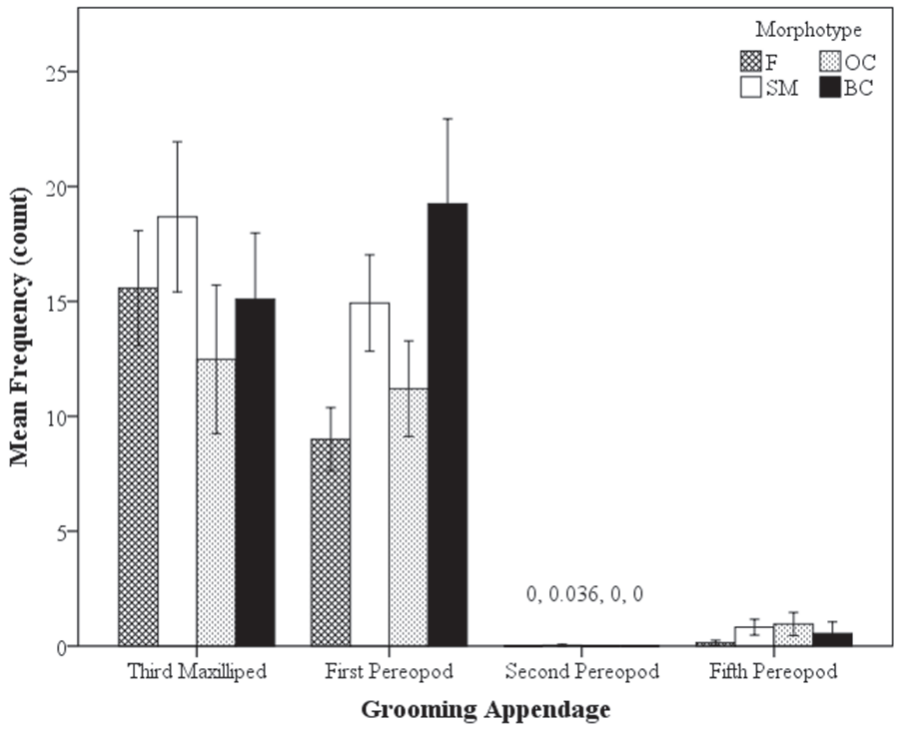
Mean frequency of use of grooming appendages of *Macrobrachium rosenbergii* females (N=21) and male morphotypes (SM: N=28, OC: N=25, BC: N=20) in 30-min time period (mean ± SE). Note: BC = blue-clawed males; F = females, OC = orange-clawed males; SM = small males.

Overall, the most frequently used grooming appendages for all individuals (females and the male morphotypes) were the M3 and P1, but there were no significant differences in the frequency of use among the grooming appendages among all three male morphotypes and females. The M3 and P1 were used equally among all groups (Kruskal-Wallis, H=3.55, P<0.001; Fig. 4). The P5 appendage was also used equally between females and the male morphotypes (Mann-Whitney U, z=-0.50, P=0.62; Fig. 4). The P2 appendage was the least used appendage for females and male morphotypes (Fig. 4).

##### Frequency of Groomed Body Parts: Overall

**Figure 5.**
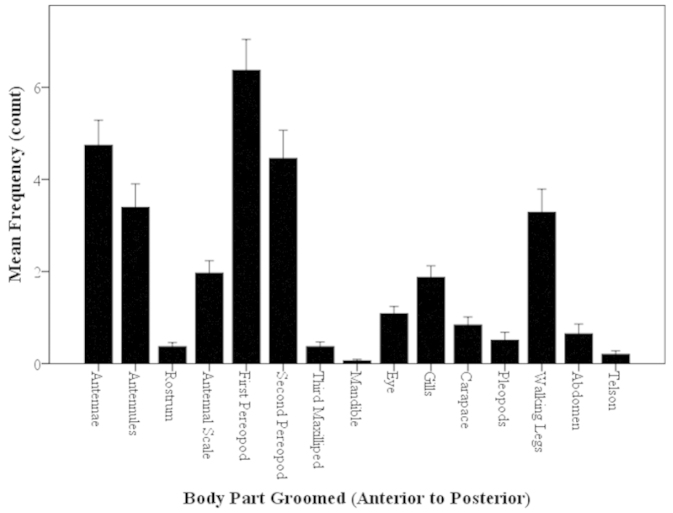
Mean frequency of body parts groomed of *Macrobrachium rosenbergii* (N=94) in a 30-min time period (mean ± SE). Body parts in graph labeled from anterior to posterior, left to right. Note: no significant differences between the two highest body parts, p>0.05.

Grooming of the body parts in terms of frequency were analyzed. The total grooming actions in terms of frequency for all observations (N=94) was 2,838 behaviors. Common grooming behaviors observed include M3 scraping the sensory structures (A1 and A2), P1 brushing the respiratory structures (enclosed gills) and general body grooming by the pereiopods. There was no difference in use of a single appendage between the females and male morphotypes. The most frequently groomed part of the body was the first pereopods (P1) (which are also frequently used grooming appendages) (Fig. 5). As the P1 appendage is a commonly used grooming appendage, it may accumulate fouling material, thus it must be important to keep free of fouling. Although the P1 was the most frequently groomed body part (by the M3 and opposite P1 appendage), there was no significant difference in the frequency of use between the P1 and the next most frequently groomed body area, the antennae (Kruskal-Wallis, H=420.73, P<0.001; Mann-Whitney U, z=-1.83, P=0.067; Fig. 5). The third most frequently groomed area of the body (3^rd^ highest) is the second pereopod (P2) (Fig. 5). The P2 may not be a frequently used as a grooming appendage, but it may be important to keep free of fouling as it is frequently groomed. There was no preference or correlation among the frequencies of grooming parts and location (anterior or posterior body parts) (Mann-Whitney U, z=-0.41, P=0.68; Fig. 5).

##### Frequency of Groomed Body Parts: Morphotypes

**Figure 6.**
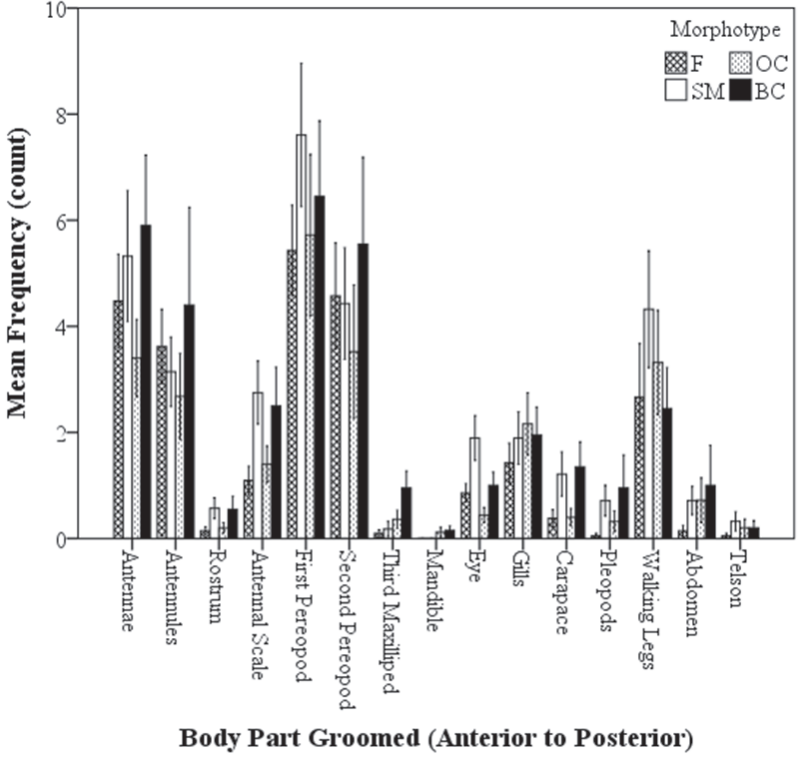
Mean frequency of body parts groomed of *Macrobrachium rosenbergii* females (N=21) and male morphotypes (SM: N=28, OC: N=25, BC: N=20) in a 30-min time period (mean ± SE). Body parts in graph labeled from anterior to posterior, left to right. Note: BC = blue-clawed males; F = females, OC = orange-clawed males; SM = small males.

**Figure 7.**
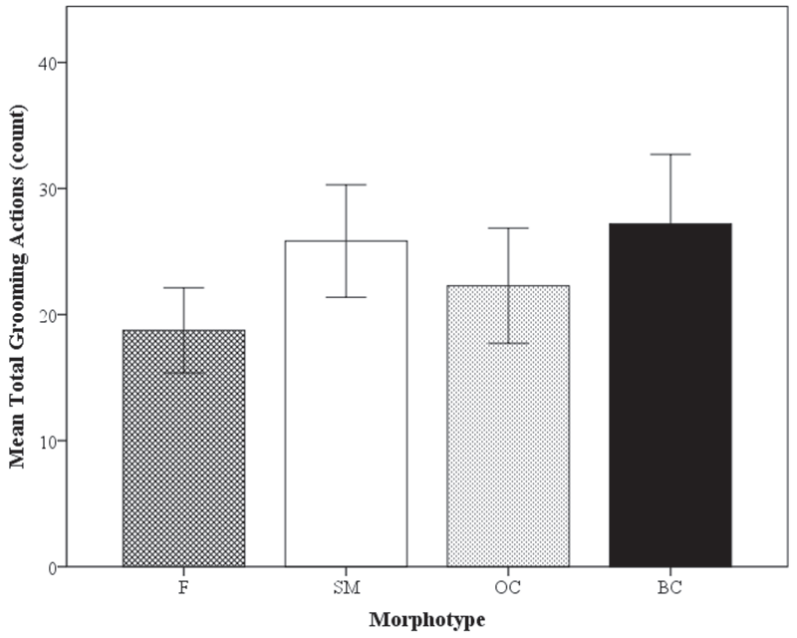
Mean total frequency of grooming behaviors of *Macrobrachium rosenbergii* females (N=21) and male morphotypes (SM: N=28, OC: N=25, BC: N=20) in 30-min time period (mean ± SE). Note: BC = blue-clawed males; F = females, OC = orange-clawed males; SM = small males. No significant differences between the groups, p>0.05.

Important sensory, locomotive, and morphological areas of the body were selected among the females and male morphotypes to determine if these areas were groomed equally. These areas and functionality include (1) the walking legs (P5–P8) (locomotive), (2) antennal scale (precision in agonistic interactions and steering and braking function) and (3) pleopods (reproduction and forward swimming). There were no significant differences in the grooming frequency of these selected areas for the females and all male morphotypes (i.e. frequency of grooming antennal scale of females and BC males were equal) (Mann-Whitney U, z=-1.41–0.77, P=0.16–0.97; Fig. 6). There were no significant differences in the mean total frequency of grooming actions for females and male morphotypes (Mann-Whitney U, z=-0.64–0.40, P=0.52–0.85; Fig. 7). All types of individuals had statistically the same number of grooming actions in 30-min trials (Fig. 7).

##### Time Spent on Body Parts: Overall

**Figure 8.**
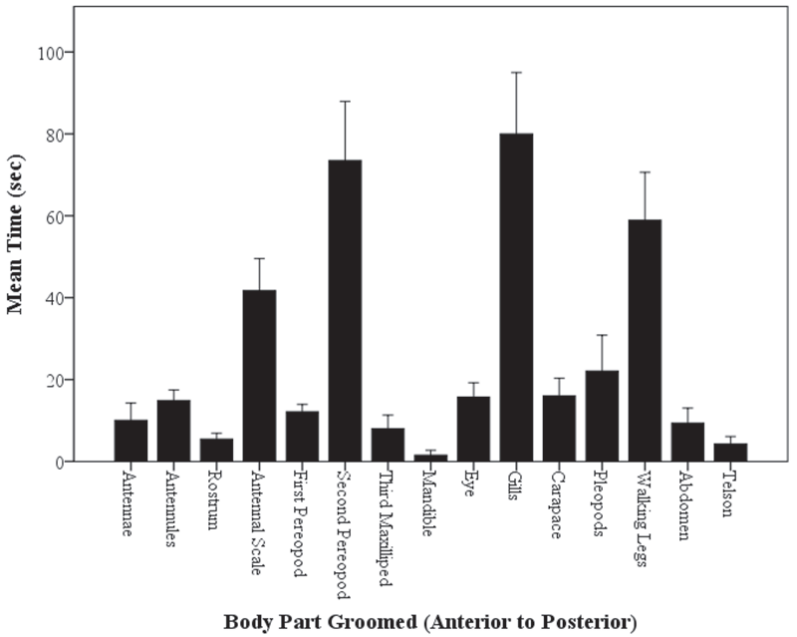
Mean time (sec) of body parts groomed of *Macrobrachium rosenbergii* (N=94) in a 30-min time period (mean ± SE). Body parts in graph labeled from anterior to posterior, left to right. Note: no significant differences between the two highest body parts, p>0.05.

Although a part may be frequently groomed, it may not be groomed for a long amount of time. The amount of time spent grooming body parts was analyzed. In all 94 observations, the total time spent observing individuals was 47 hrs. Of those 47 hours, the total time spent grooming by all 94 shrimps was 35,132 sec (9.76 hrs). The part groomed for the longest average time was the gills (Fig. 8), which was not a frequently groomed body part (Fig. 5, 6). The body area groomed for the second highest time was the second pereopods (P2), but there was no significant difference between the time spent grooming these two parts (gills and P2) (Kruskal-Wallis, H=302.66, P<0.001; Mann-Whitney U, z=-0.51, P=0.6067; Fig. 8). There was no obvious correlation or preference in grooming time by location (anterior or posterior body part) (Mann-Whitney U, z=0.96, P=0.34; Fig. 8).

##### Time Spent on Body Parts: Morphotypes

**Figure 9.**
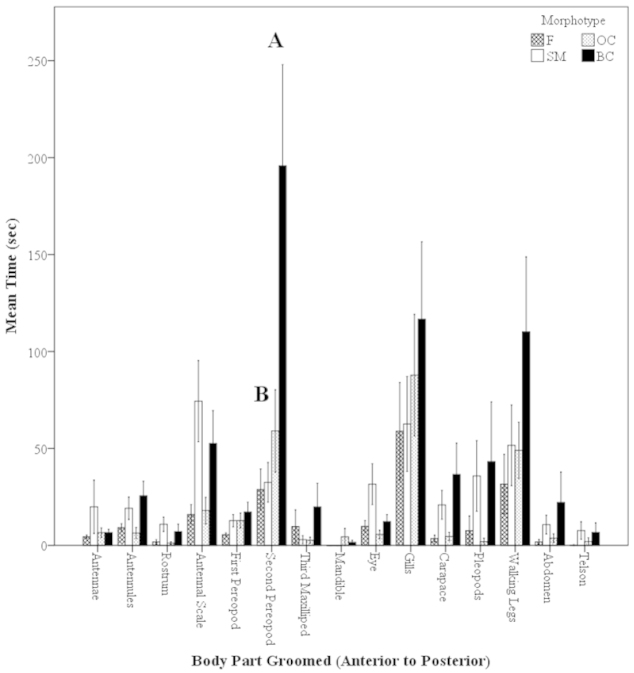
Mean time (sec) of body parts groomed of *Macrobrachium rosenbergii* females (N=21) and male morphotypes (SM: N=28, OC: N=25, BC: N=20) in a 30-min time period (mean ± SE). Body parts in graph labeled from anterior to posterior, left to right. Note: BC = blue-clawed males; F = females, OC = orange-clawed males; SM = small males. Different letters indicate significant differences among body parts (B is referring to the females and SM and OC males).

**Figure 10.**
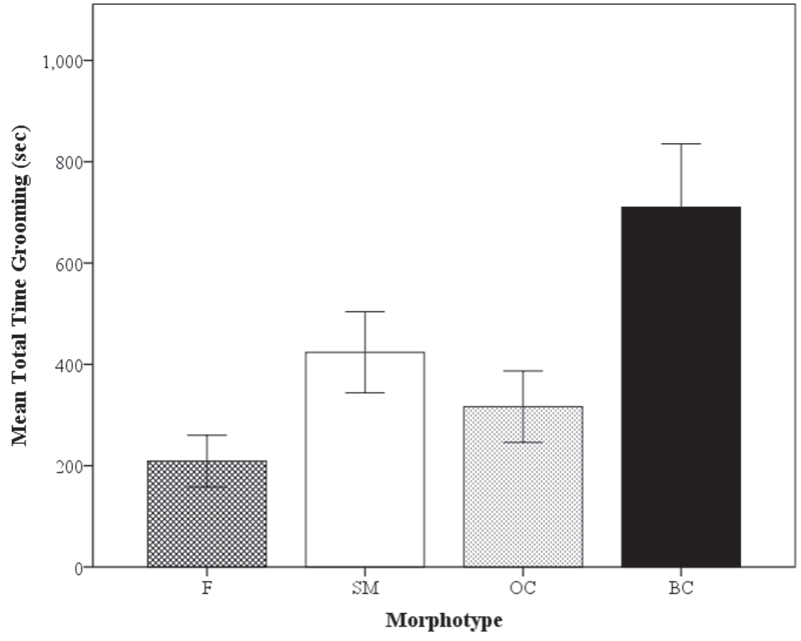
Mean total time (sec) spent grooming of *Macrobrachium rosenbergii* females (N=21) and male morphotypes (SM: N=28, OC: N=25, BC: N=20) in 30-min time period (mean ± SE). Note: BC = blue-clawed males; F = females, OC = orange-clawed males; SM = small males. No significant differences between the groups, p>0.05.

When looking at the time spent grooming different body parts in the morphotypes, there were clear differences. There were significant differences in the time spent grooming the second pereopods (P2) between the BC males and all other groups (OC and SM males as well as F (Kruskal-Wallis, H=8.72, P=0.033; Mann-Whitney U, z=-2.73 to -2.02, P=0.006–0.044; Fig. 9). The large BC males spent significantly more time grooming the P2 than all other male morphotypes (Fig. 9). BC males spent the most time grooming most areas of the body (ten out of fifteen body parts; except the antennae, rostrum, mandible, eye and telson) (Fig. 9). In all five cases in which the BC males did not have the highest average time, the SM males spent the most time grooming those areas (Fig. 9). Similarly to the grooming of the body parts in terms of frequency, areas of the body considered important in sensory, locomotive, and morphological functions were selected and analyzed in terms of time: walking legs, the antennal scale and pleopods. There were no significant differences in the time spent grooming these areas among the females and all male morphotypes (i.e. female antennal scale is equal to BC male antennal scale) (Kruskal-Wallis, H=2.00–9.51, P=0.023–0.57; Mann-Whitney U, z=-1.21–0.96, P=0.23–0.92; Fig. 9). There were no significant differences in the mean total time in 30-min trial spent on grooming activities for females and male morphotypes (Mann-Whitney U, z=-1.88–1.11, P= 0.06–0.97; Fig. 10). However, there seems to be a trend for BC males to spend more time grooming compared to other male morphotypes and females, but it is not significant; females and male morphotypes spent about the same amount of total time grooming (Fig. 10).

##### Time Budget

Overall in *Macrobrachium rosenbergii*, a generous proportion of time is spent grooming the body. The average time budget for grooming was 19.3%, indicating up to one-fifth of *Macrobrachium rosenbergii*’s time may be dedicated to grooming when primary behaviors (fighting, mating, etc.) are not present.

**Figure 11.**
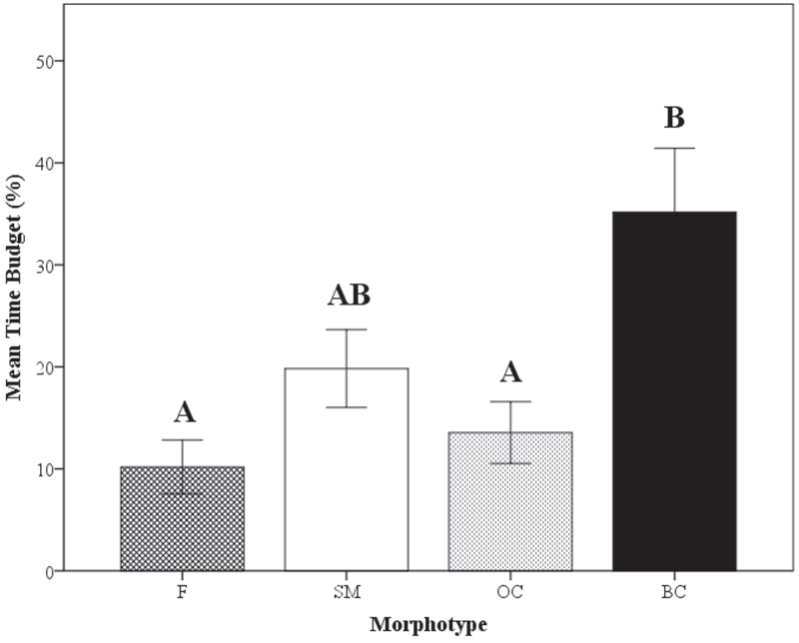
Mean time budget for grooming of *Macrobrachium rosenbergii* morphotypes (F: N=21, SM: N=28, OC: N=25, BC: N=20) in 30-min time period. Overall mean time budget of species is 19.3%. Note: BC = blue-clawed males; F = females, OC = orange-clawed males; SM = small males. Different letters indicate significant differences among groups.

Of the females and male morphotypes, the BC males had the highest average time budget for grooming, 35.2%, which was significantly higher than that of females (10.2%) (Mann-Whitney U, z=-2.93, P=0.0033; Fig. 11) and OC males (13.6%) (Mann-Whitney U, z=2.69, P=0.0072; Fig. 11). The grooming time budget for females, SM males and OC males were 10.2%, 19.8% and 13.6%, respectively (Fig. 11).

#### Social Grooming Observations (Observations #2)

In all social observations (N=8), not one grooming behavior occurred. Hence, the observations were ended prematurely at a lower sample size compared to the other observations (Observations #1 and #3). The shrimps were in a social situation where many behaviors such as searching for mates, displaying, touching and grooming can occur. The grooming time budget was 0% for all observations.

#### Agonistic Grooming Observations (Observations #3)

**Figure 12.**
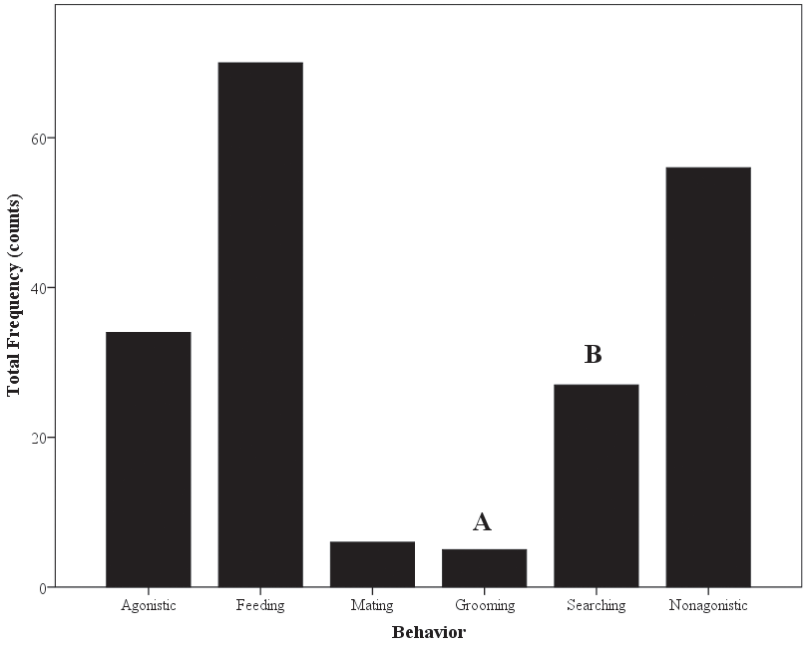
Mean frequency of behaviors during agonistic observations (N=20) of *Macrobrachium rosenbergii*. Note: Different letters indicate significant differences among morphotypes. Note: different letters indicate significant differences among behaviors.

The paired shrimps (N=20; Table 1) physically interacted often (Fig. 12). No social grooming (allogrooms) occurred in any trial. The most frequent behavior was feeding and the least common behavior was grooming, making up 35% and 2.5% of the grooming activites, respectively (Fig. 12). While there was no food given, shrimps picked up particulate matter in the water column that had settled on the bottom. Some examples of non-agonistic interactions that occurred include cheliped or antennae touching and antennal flicking. There was a significant difference in the frequency of these behaviors, with grooming occurring significantly less than all other behavioral categories except mating (Mann-Whitney U; z=-3.52; P<0.001; Chi-squared test; χ^2^=104.5; P<0.001; Fig. 12). Mating was not compared to grooming frequencies due to mating only possible in a fraction of the treatments where females and males were present (Treatments 4, 7 and 9; Table 1).

**Figure 13.**
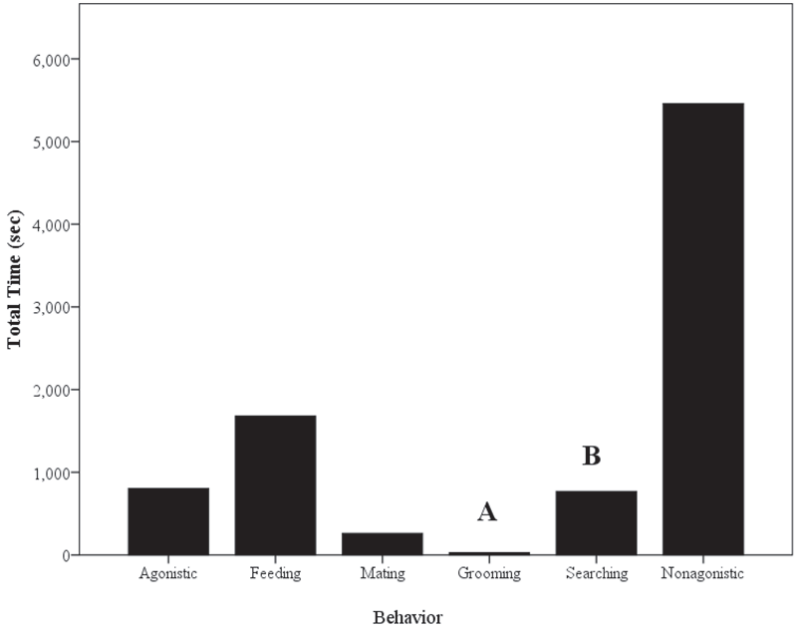
Mean time (sec) spent on behaviors during agonistic observations (N=20) of *Macrobrachium rosenbergii*. Note: different letters indicate significant differences among behaviors.

The behavior that occurred for the longest time was non-agonistic interactions and grooming occurred for the shortest amount of time (Fig. 13). There were significant differences in the time spent among the various behaviors with grooming lasting significantly less time than all other behaviors except mating (Mann-Whitney U; z=-4.00; P<0.001; Chi-squared test; χ^2^=13,610.7; P<0.001; Fig. 13). The grooming time budget was 0.31% in these treatments (N=20).

## Discussion

### Grooming in *Macrobrachium rosenbergii*

Overall, *Macrobrachium rosenbergii* showed similar grooming behaviors compared to other caridean shrimps including the usage of specific grooming appendages (third maxillipeds, first, second and fifth pereopods) (Bauer 1978, 1981, Felgenhauer and Schram 1979); priority of body parts groomed (ie: P1 groom the gills for respiration, M3 scrape the antennules for sensory reception) (Bauer 1977, 1979) and time budget dedicated to grooming activities (Table 2). Each day shrimps spend a large portion of their time grooming, leaving fewer hours to accomplish other activities such as finding suitable habitat, maintaining and defending that habitat, resting, reproduction, and searching for food. The amount of time caridean shrimps spend grooming suggests there must be an important benefit for the activity. Bauer (1979, 1989, 2004) has shown that grooming is an adaptive behavior especially in caridean crustaceans due to the development of complex structures and behaviors related to grooming.

**Table 2.** Grooming time budgets of caridean shrimps.

Species	Grooming Time Budget (%)	Presence of Conspecifics?	Reference
*Heptacarpus pictus*	27%	No	Bauer 1977
*Macrobrachium grandimanus*	25%	No	VanMaurik and Wortham 2011
*Macrobrachium rosenbergii*	19%	No	Current study (Observations #1)
*Macrobrachium rosenbergii*	0%	Partial	Current study (Observations #2)
*Macrobrachium rosenbergii*	0.31%	Yes	Current study (Observations #3)

Although there were similarities in the grooming behaviors of *Macrobrachium rosenbergii* and other carideans, the male morphotypes of *Macrobrachium rosenbergii* differed in grooming behaviors. These morphotypes are known to differ in their behavior based on their niche in the social hierarchy, and therefore it is plausible their grooming behaviors and time dedicated to grooming may differ (Ra’Anan and Sagi 1985). BC males spent a significantly longer time grooming the second pereopods compared to all other males and females, indicating this area may be important for this hierarchical group. BC males are the terminal molt stage and are not able to rid of fouling by molting (Amir Sagi, personal communication). The second pereopods of BC males are used to dominate other males, as well as for protection and defense of females. As a result, BC males seem to invest much time and energy to remove fouling.

The SM and BC male morphotypes differ in their behavior yet they have the highest total frequency and time of grooming actions along with the highest time budgets for grooming. This may be attributed to the relative efficiencies of their grooming activities: SM males are highly mobile and may experience greater fouling pressures (Ra’Anan and Sagi 1985, Bauer 1989) and an increased grooming need, resulting in much time and effort dedicated to the removal of fouling agents. BC males have lower fouling pressures due to their limited mobility but higher pressures as they are unable to molt and rid of fouling compared to SM males. Grooming in BC males may not be very efficient due to cumbersome grooming appendages, as grooming frequently occurs (Ra’Anan and Sagi 1985). It is also likely that BC are equally efficient at grooming but have a higher need for grooming since they are the terminal molt stage.

Although BC males have the highest grooming time budget, it appears that most of this time is spent in the grooming of the P2 appendage. The BC males groomed the P2 appendage frequently and for a long time, which may be due to the setal patch located on the propodus. The setal patch may participate in displays to ward off other males from territories (Correa et al. 2000). The P2 appendage is also used in the protection and defense of females. The fact that the BC males dedicate much time to the grooming of this appendage indicates it is may be important in maintaining the dominance position of these males in the social hierarchy.

We found that primary (higher priority) behaviors such as feeding and defense are of greater importance and should occur more frequently than grooming when primary behaviors are possible. Grooming was absent or rare when primary actions occurred (social grooming observations, Observations #2), therefore grooming should be considered as a secondary behavior, as hypothesized by Bauer (1989). Females with embryos were not used in grooming observations; the time spent on pleopod grooming would likely be higher if they were included (Bauer 1979). When individuals were in contact with other conspecifics, primary actions such as fighting, feeding, mating, searching and non-agonistic interactions occurred more often and longer, taking precedence over grooming (Table 2). Although grooming is likely of lower priority than primary actions, it is still an important adaptive behavior to fouling pressures and it maintains vital, primary functions such as locomotion, respiration, chemosensory reception and reproduction.

## Conclusions

As hypothesized, *Macrobrachium rosenbergii* showed similar grooming behaviors and grooming time budgets compared to other caridean shrimps indicating grooming behaviors have evolved in response to the fouling pressures experienced in an aquatic environment. We found that the BC males dedicate much time to the care of the P2 appendages which are used in displays, protection and defense. This behavior contributes to the high time budget for grooming in the BC males. Grooming was also hypothesized to be a secondary behavior, only occurring when other behaviors are not essential (Bauer 1989). The results of this study indicate grooming in shrimps is a secondary behavior as demonstrated by the behaviors of the commercially important shrimp, *Macrobrachium rosenbergii*.

*Macrobrachium rosenbergii* is frequently grown in aquaculture (New and Nair 2012) with the BC males being the largest in size and most valuable in terms of yield and profit, even though all individuals (SM, OC, BC and females) are grown and may be sold for consumption (New and Valenti 2000, FAO 2004). Once these shrimps reach a certain age or size, they are usually exported to an outdoor grow-out tank, which is subject to agricultural or industrial runoff, sedimentation, algal blooms and fouling organisms such as bacteria or invertebrates (New and Valenti 2000, Bauer 2002). As a result, fouling is very likely high, leading to survival implications (decreased respiration, movement, sensory reception or defense) during the intermolt period. The best economic interest of aquaculture farmers should be to have the lowest mortality rate of shrimps in order to allow them to grow to the largest, most profitable size since all BC males must survive past the SM and OC male phase to develop until the most profitable BC male. Besides water quality and filtration, the population density in aquaculture tanks is an important factor regulating the behaviors of shrimps (high densities correlate with greater occurrence of primary behaviors such as mating and defense). Therefore, yields depend on the size of the hierarchial groups in the grow-out tanks or ponds. Grooming behaviors are important to understand as antifouling adaptation, particularly in high-density populations such as aquaculture settings.
